# Prevalence and correlates of vitamin D status in African American men

**DOI:** 10.1186/1471-2458-9-191

**Published:** 2009-06-18

**Authors:** Marilyn Tseng, Veda Giri, Deborah W Bruner, Edward Giovannucci

**Affiliations:** 1 Cancer Prevention and Control Program, Fox Chase Cancer Center, Philadelphia, PA 19111, USA; 2 Department of Kinesiology, California Polytechnic State University, San Luis Obispo, CA 93407, USA; 3 School of Nursing, University of Pennsylvania, Philadelphia, PA 19104, USA; 4 Department of Nutrition, Harvard School of Public Health, Boston, MA 02115, USA; 5 Department of Epidemiology, Harvard School of Public Health, Boston, MA 02115, USA

## Abstract

**Background:**

Few studies have examined vitamin D insufficiency in African American men although they are at very high risk. We examined the prevalence and correlates of vitamin D insufficiency among African American men in Philadelphia.

**Methods:**

Participants in this cross-sectional analysis were 194 African American men in the Philadelphia region who were enrolled in a risk assessment program for prostate cancer from 10/96–10/07. All participants completed diet and health history questionnaires and provided plasma samples, which were assessed for 25-hydroxyvitamin D (25(OH)D) concentrations. We used linear regression models to examine associations with 25(OH)D concentrations and logistic regression to estimate odds ratios (OR) for having 25(OH)D ≥ 15 ng/mL.

**Results:**

Mean 25(OH)D was 13.7 ng/mL, and 61% of men were classified as having vitamin D insufficiency (25(OH)D <15 ng/mL). Even among men with vitamin D intake ≥ 400 IU/day, 55% had 25(OH)D concentrations <15 ng/mL. In multivariate models, 25(OH)D concentrations were significantly associated with supplemental vitamin D intake (OR 4.3, 95% confidence interval (CI) 1.5, 12.4) for >400 vs. 0 IU/day), milk consumption (OR 5.9, 95% CI 2.2, 16.0 for ≥ 3.5 vs. <1 time per week), and blood collection in the summer. Additionally, 25(OH)D concentrations increased with more recreational physical activity (OR 1.3, 95% CI 1.1, 1.6 per hour). A significant inverse association of body mass index with 25(OH)D concentrations in bivariate analyses was attenuated with adjustment for season of blood collection.

**Conclusion:**

The problem of low vitamin D status in African American men may be more severe than previously reported. Future efforts to increase vitamin D recommendations and intake, such as through supplementation, are warranted to improve vitamin D status in this particularly vulnerable population.

## Background

In addition to its association with bone loss, fracture, osteomalacia, and other skeletal conditions, suboptimal concentrations of circulating 25(OH)D have been linked to non-skeletal chronic diseases, including cancer, diabetes, heart disease, autoimmune conditions, hypertension, andinfection [1-3]. Because the skin pigment melanin absorbs sunlight [4], the most important source of vitamin D [5], black adults are at particularly high risk for vitamin D insufficiency. Few studies, however, have examined vitamin D insufficiency in African American men, particularly in a northern climate that may place them at even higher risk [6,7]. We examined the prevalence and correlates of vitamin D insufficiency in a sample of community-dwelling African American men in the Philadelphia region (40° 00' latitude).

## Methods

### Study sample

Participants in the study were African American men enrolled in the Fox Chase Cancer Center (FCCC) Prostate Cancer Risk Assessment Program (PRAP). PRAP was established by FCCC investigators in 1996 to offer education and preventive interventions to men at potentially high risk for prostate cancer, and to serve as a research base for studying gene-environment interactions in prostate cancer [8]. African American men were eligible for the program if they were between 35 and 69 years of age and had no history of prostate cancer. Recruitment strategies included referrals from prostate cancer patients at FCCC, radio and newspaper advertisements, and physician and self-referrals.

Of 440 African American men enrolled in PRAP between October, 1996, and October, 2007, we excluded those who were recruited through satellite locations and whose plasma samples thus could not be processed immediately after collection (N = 79); who did not provide a blood sample after July, 2000, when standard procedures were established for processing and storing plasma samples in the FCCC Biosample Repository (N = 72); or who had a previous diagnosis of cancer (N = 5). In addition, because the analysis was nested within a study of dietary intake and vitamin D status, men who did not return a food frequency questionnaire (N = 73) or who reported an infeasible energy intake (<500 kcal/day or >4400 kcal/day) (N = 17) were excluded, leaving 194 men available for these analyses.

Participants provided their written, informed consent to participate in study procedures. All procedures were in accordance with institutional ethical standards, and the project was approved by the Institutional Review Board at the Fox Chase Cancer Center.

### Data collection

Upon enrollment into PRAP, men completed a health history questionnaire and the Harvard Diet Assessment Form [9]. At initial and follow-up appointments, participants also contributed 51 mL of blood for storage in the FCCC Biosample Repository. The health history questionnaire elicited information on sociodemographic characteristics, occupation, family history of prostate cancer, smoking status, physical activity, and self-reported height and weight. Occupational sun exposure was assessed based on each participant's reported occupation, which was classified into one of three categories according to presumed sunlight exposure (indoor work, combined indoor and outdoor work, outdoor work), using an index that was related to mortality from non-melanoma skin cancer in previous studies [10-12]. The Diet Assessment Form elicited information on frequency of intake of 126 food items, and on length of use and dosage of dietary supplements.

### Assessment of plasma 25(OH)D concentration

Concentrations of plasma 25(OH)D were determined by Heartland Assays, Inc. (Ames, IA) with a direct, competitive chemiluminescence immunoassay (CLIA) using the DiaSorin LIAISON platform [13]. The assay is co-specific for 25-hydroxyvitamin D_3 _and 25-hydroxyvitamin D_2_, so that total 25(OH)D is reported. Inter-assay variability estimated from other samples in the same laboratory ranged from 12.7–13.6%, and intra-assay variability ranged from 9.3–11.0% (personal communication).

### Statistical analyses

We used linear regression models to examine associations of sociodemographic, lifestyle, and dietary factors with 25(OH)D concentration, and logistic regression models to estimate odds ratios for having 25(OH)D concentrations ≥ 15 ng/mL). Cutpoints to define vitamin D insufficiency vary. We chose a cutpoint of 15 ng/mL to define the lower limit of the normal range of 25(OH)D concentration [5]. The same cutpoint has been used as a criterion to define low vitamin D status in other studies [14-16], including recent studies conducted among African American men [17,18]. While 30–32 ng/mL (75–80 nmol/L) are other common cutpoints for optimal vitamin D concentrations with respect to a variety of health outcomes [16,19], too few men in our sample (N = 2) had concentrations above 30 ng/mL to permit a sufficiently powered analysis.

Fortified foods such as milk (~100 IU/cup) and cereal (often ~40 IU/cup) are the primary sources of vitamin D in the United States [5] and were therefore considered as predictors of vitamin D status. Milk intake was calculated as the sum of frequencies of intake of skim or low fat milk and whole milk. Intakes of alcohol, fish, and eggs were not associated with 25(OH)D levels in bivariate analyses and were not considered further.

Categorical covariates were coded using dummy variables to allow for non-linear associations across categories. Variables were included as potential confounders in multivariate models if they were significantly associated with 25(OH)D concentrations in unadjusted linear or logistic regression models. Variables that were not significant predictors in linear or logistic regression models were subsequently dropped. All statistical analyses were conducted using SAS for Windows version 9.1.2 (Cary, NC).

## Results

Among the 194 African American men in our sample, mean (SD) age was 49.6 (8.4) years, and most men were either overweight (46% with body mass index (BMI) 25–<30 kg/m^2^) or obese (39% with BMI ≥ 30 kg/m^2^) (Table 1). With respect to vitamin D status, only 39% had 25(OH)D concentrations ≥ 15 ng/mL, while 27% had concentrations of 10–<15 ng/mL, and 34% had concentrations <10 ng/mL (Table 1). Only two men had concentrations >30 ng/mL.

**Table 1:** Distribution of descriptive characteristics in 194 African American men^1^, and associations with 25(OH)D concentrations.

	Distribution (%)	Mean 25(OH)D (ng/mL)^2^	25(OH)D <15 ng/mL (%)
Plasma 25(OH)D (ng/mL)		--	--
Mean (SD)	13.7 (6.1)		
<10	34		
10–<15	27		
≥ 15	39		
			
Age (years)			
Mean (SD)	49.6 (8.4)		
35–44	29	13.1	65
45–54	45	13.4	61
55–74	26	14.7	58
			
Level of education			
high school graduate	21	13.8	56
some college	42	13.5	63
college graduate	22	14.3	57
graduate degree	15	13.1	69
			
Body mass index (kg/m^2^)			
Mean (SD)	29.6 (5.4)		
<25	15	15.0^a^	50
25–<30	46	14.0^ab^	57
30–<35	26	13.2^ab^	67
≥ 35	13	11.5^b^	76
			
Recreational physical activity (hours/week)			
Mean or median	2.3 (2.1)		
≤ 1	42	12.7^a^	74^a^
>1–3	34	14.6^b^	48^b^
>3	24	15.4^b^	45^b^
			
Occupational sun exposure			
Indoor	35	13.9^ab^	58
Mixed/Outdoor	18	12.0^a^	68
Unknown occupation	28	13.2^ab^	67
Not currently working	20	15.5^b^	53
			
Smoking status			
Never	62	14.2	57
Former	23	13.5	69
Current	15	12.0	67
			
Total vitamin D (IU/day)			
Mean (SD)	371 (331)		
<200	45	12.2^a^	68
200–<400	21	14.4^ab^	58
≥ 400	35	15.0^b^	55
			
Dietary vitamin D (IU/day)			
Mean (SD)	181 (114)		
<200	70	13.4	64
200–<400	24	14.2	59
≥ 400	7	14.3	46
			
Milk intake (8 oz glasses/week)			
Median (1^st^, 3^rd ^quartile)	1 (0.5–3)		
<1	44	13.0	69^a^
1–<3.5	38	13.7	60^ab^
≥ 3.5	18	15.2	46^b^
			
Cereal intake (cups/week)			
0	22	12.6^a^	62
≤ 1	41	13.1^ab^	67
>1	37	14.9^b^	54
			
Supplemental vitamin D (IU/day)			
0	55	11.9^a^	73^a^
≤ 400	32	15.8^b^	46^b^
>400	13	15.8^b^	52^b^
			
Season of blood collection			
Summer	26	18.0^a^	31^a^
Fall	24	13.8^b^	61^b^
Winter	29	10.3^c^	80^c^
Spring	21	12.7^b^	73^b^

^1 ^Due to missing values, N = 187 for BMI, N = 136 for recreational physical activity, and N = 180 for smoking status.

^2 ^Differences are statistically significant (p < 0.05) between categories with different superscripts. Analysis-of-variance was used to compare mean 25(OH)D across categories, with least square means used in post-hoc comparisons. The Cochran-Mantel-Haenszel test was used to compare proportions with 25(OH)D <15 ng/mL across categories.

In unadjusted, bivariate analyses, 25(OH)D concentrations were higher with lower BMI and with more recreational physical activity. Mean 25(OH)D was 15.0 ng/mL among non-overweight men vs. 11.5 ng/mL among men with BMI ≥ 35 kg/m^2^. It was 12.7 ng/mL among men who exercised ≤ 1 hour/week vs. 15.4 ng/mL among men who exercised >3 hours/week. Concentrations were also higher with greater milk and cereal intake and with supplemental vitamin D intake, although concentrations and level of insufficiency were similar regardless of level of supplementation. Notably, even among men consuming at least 400 IU of vitamin D per day, the recommended intake for men 51–70 years of age [5], mean 25(OH)D was only 15.0 ng/mL, and 55% had concentrations <15 ng/mL.

Vitamin D concentrations varied with season as well, with the highest mean concentration (18.0 ng/mL) and lowest prevalence of insufficiency (31%) occurring during the summer, and the lowest mean concentration (10.3 ng/mL) and highest prevalence of insufficiency (80%) occurring during the winter. Additionally, vitamin D concentrations were lowest in men with occupations categorized as being outdoor or mixed indoor/outdoor, and highest in men not currently working. We observed non-significant trends of increasing 25(OH)D with age, and lower 25(OH)D among current smokers.

In multivariate models, 25(OH)D concentrations were significantly associated with supplemental vitamin D intake and milk consumption, and significantly inversely associated with blood collection in fall, winter, or spring vs. summer (Table 2). In additional multivariate analyses limited to 136 men not missing data on recreational physical activity, 25(OH)D levels increased by 0.38 ng/mL with every additional hour per week of physical activity (Table 2). Notably, unadjusted, bivariate associations of 25(OH)D concentrations with BMI and occupational sun exposure were attenuated with adjustment for season; mean BMI was significantly (p = 0.006) higher during winter (31.2 kg/m^2^) than during summer (28.2 kg/m^2^) months, and men with occupational sun exposure categorized as being mixed or outdoor were more likely to have contributed their samples during winter (32%) than during summer (12%) months.

**Table 2:** Multivariate adjusted betas and odds ratios (OR) with 95% confidence intervals (CI) for 25(OH)D ≥ 15 ng/mL.

	Multivariate^1 ^model (N = 194)		Multivariate model + physical activity^2 ^(N = 136)	
	Beta^3 ^(p-value)	OR (95% CI)	Beta^3 ^(p-value)	OR (95% CI)
Supplemental vitamin D (IU/day)				
0	ref	1.0	ref	1.0
≤400	4.5 (<0.0001)	5.9 (2.6, 13.5)	4.3 (<0.0001)	8.1 (2.9, 22.6)
>400	4.5 (<0.0001)	4.3 (1.5, 12.4)	4.5 (0.001)	4.8 (1.3, 18.6)
				
Milk intake (times/week)				
<1 time/week	ref	1.0	ref	1.0
1–<3.5 times/week	1.4 (0.07)	2.3 (1.0, 5.2)	1.7 (0.08)	2.8 (1.0, 7.5)
≥3.5 times/week	3.3 (0.001)	5.9 (2.2, 16.0)	3.1 (0.01)	6.6 (1.9, 23.6)
				
Season of blood collection (%)				
Summer	ref	1.0	ref	1.0
Fall	-4.70 (<0.0001)	0.2 (0.1, 0.5)	-4.34 (0.0004)	0.2 (0.1, 0.7)
Winter	-8.59 (<0.0001)	0.05 (0.02, 0.1)	-8.56 (<0.0001)	0.04 (0.01, 0.2)
Spring	-6.22 (<0.0001)	0.1 (0.03, 0.2)	-5.69 (<0.0001)	0.1 (0.03, 0.4)
				
Recreational physical activity (hours/week)	--	--	0.38 (0.07)	1.3 (1.1, 1.6)
R^2^	0.40		0.39	

^1 ^Model including supplemental vitamin D, milk intake, and season of blood collection.

^2 ^Model including supplemental vitamin D, milk intake, season of blood collection, and recreational physical activity.

^3 ^Betas represent absolute mean change in 25(OH)D concentration relative to referent category. For physical activity, beta represents absolute mean change in 25(OH)D concentration for each one-hour increase in recreational physical activity per week.

## Discussion

We noted very low concentrations of 25(OH)D and a very high prevalence of insufficiency (61%) in a sample of adult African American men in the Philadelphia region, indicating a more severe problem of hypovitaminosis D than has been reported previously among African American men [6,7,17,18,20,21]. Comparison with previous studies is difficult because different cutpoints have been used to define insufficiency, and also because we did not use an external control to permit comparison with results from other laboratories. The lower mean 25(OH)D concentration in our sample than in African American men in Massachusetts (17–25 ng/mL) [6,7] and Washington, DC (18 ng/mL) [18] may be due to variability in methods [22,23]. However, mean 25(OH)D concentration during summer months in our sample is only slightly lower than the mean of ~20 ng/mL estimated for black participants in the Third National Health and Nutrition Examination Survey, which was conducted during the summer in northern states and during the winter in southern states [21,24]. It is comparable to mean concentrations estimated for black adults in regions with greater sun exposure – in particular, Arizona (18.2 ng/mL) [20], California (18.1 ng/mL) [25], and the South (19.0 ng/mL) [17]. This context supports the plausibility of the low vitamin D status observed in our sample.

We found large differences in vitamin D status by season, confirming the substantial contribution of season, particularly winter, to severe vitamin D insufficiency. We also found an association of physical activity with vitamin D status but could not assess whether the association could be attributed to sun exposure with outdoor activity [26-28]. The significant associations for intakes of supplemental vitamin D and milk indicate the importance of measures to increase vitamin D intake to improve vitamin D status. In multivariate analyses, vitamin D supplement use increased mean 25(OH)D concentrations by 4.5 ng/mL, and milk consumption of at least 3.5 times per week increased concentrations by 3.3 ng/mL relative to consumption of less than once per week. However, even among men with total (dietary and supplemental) vitamin D intake of >400 IU/day, the recommended intake for men over 50 years of age [5], 55% had 25(OH)D concentrations <15 ng/mL, suggesting that current dietary recommendations are not adequate to achieve optimal vitamin D concentrations in this population.

The significant association between BMI and 25(OH)D concentrations in bivariate analyses was attenuated after adjustment for season of blood draw. In our sample, BMI was significantly higher in the winter (31.2 kg/m^2^) than in the summer (28.2 kg/m^2^), a phenomenon that has been noted in other studies [29,30]. The lack of association between BMI and 25(OH)D concentrations in our sample differs from previous studies, conducted primarily in white populations, that have noted an inverse association between the two [31]. Other evidence exists to suggest that adiposity may not be as strong a predictor of low vitamin D status among African Americans as it is in whites [14,32,33].

A limitation of our study is that detailed information on sun exposure, such as time spent outdoors, sunscreen use, and other sun protection behaviors, was not available. Additionally, 21% (N = 40) of our sample completed their questionnaires over a year before collection of their blood sample used in these analyses; however, analyses excluding these men produced no meaningful differences from results based on the full sample. Self-selection into the high risk program may have biased our estimates or may limit generalizability of our results, but African American men were required to meet no eligibility criteria to enroll in the program other than having no personal history of prostate cancer. Further, we saw no association of family history of prostate cancer with 25(OH)D levels (data not shown), nor is there reason to expect that self-selection into the program would be related to major determinants of vitamin D status. Our analysis included only 194 of the 440 African American men enrolled in PRAP. Men in our sample were more likely to be never smokers than were men not included in the analysis (62% vs. 48%), but the two groups were otherwise similar with respect to age, education level, BMI, and participation in recreational physical activity (data not shown).

## Conclusion

In our sample of African American men, we found extremely low vitamin D concentrations and high prevalence of vitamin D insufficiency, suggesting a more severe problem of poor vitamin D status in this population than has been reported previously. Other notable findings were significant associations of vitamin D concentrations with supplemental vitamin D, milk intake, and physical activity, and the lack of an association with obesity. Although African Americans are at lower risk for osteoporosis and bone fractures, emerging evidence suggests that vitamin D protects against various inflammatory and autoimmune conditions, cardiovascular disease, cancer, and infection [2,3]. Thus, severe insufficiency may manifest in the African American population as increased risk for these other conditions rather than as increased risk for skeletal conditions. Indeed, recent studies suggest that hypovitaminosis D in African Americans may contribute to their higher risk of peripheral arterial disease [34] and high blood pressure [35] relative to whites. Future efforts should focus on reconsidering intake recommendations and increasing vitamin D intake, such as through supplementation, in order to improve vitamin D status in this population.

## List of Abbreviations

25(OH)D: 25-hydroxyvitamin D; BMI: body mass index; FCCC: Fox Chase Cancer Center; PRAP: Prostate Cancer Risk Assessment Program.

## Competing interests

The authors declare that they have no competing interests.

## Authors' contributions

MT was responsible for initiating the study, analyzing the data, and drafting the manuscript. VG and DB contributed to the design of the study, provided information on the Prostate Cancer Risk Assessment Program, and contributed to drafts of the manuscript. EG contributed to the study concept, interpretation of results, and suggestions towards subsequent drafts of the manuscript. All authors read and approved the final manuscript.

## Pre-publication history

The pre-publication history for this paper can be accessed here:


